# Supplementary material for: N-glycosylation of NANOG regulates stemness and apoptosis in colon cancer cells
Source: PLoS One. 2025 Nov 20;20(11):e0336779. doi: 10.1371/journal.pone.0336779 (PMC12633897; doi:10.1371/journal.pone.0336779)
Supplement: S1 File — (DOCX) [file pone.0336779.s001.docx]

MUT-1 target sequence：

gctagcATGAGTGTGGATCCAGCTTGTCCCCAAAGCTTGCCTTGCTTTGAAGCATCCGACTGTAAAGAATCTTCACCTATGCCTGTGATTTGTGGGCCTGAAGAAAACTATCCATCCTTGCAAATGTCTTCTGCTGAGATGCCTCACACGGAGACTGTCTCTCCTCTTCCTTCCTCCATGGATCTGCTTATTCAGGACAGCCCTGATTCTTCCACCAGTCCCAAAGGCAAACAACCCACTTCTGCAGAGAAGAGTGTCGCAAAAAAGGAAGACAAGGTCCCGGTCAAGAAACAGAAGACCAGAACTGTGTTCTCTTCCACCCAGCTGTGTGTACTCAATGATAGATTTCAGAGACAGAAATACCTCAGCCTCCAGCAGATGCAAGAACTCTCCAACATCCTGgccCTCAGCTACAAACAGGTGAAGACCTGGTTCCAGAACCAGAGAATGAAATCTAAGAGGTGGCAGAAAAACAACTGGCCGAAGAATAGCAATGGTGTGACGCAGAAGGCCTCAGCACCTACCTACCCCAGCCTTTACTCTTCCTACCACCAGGGATGCCTGGTGAACCCGACTGGGAACCTTCCAATGTGGAGCAACCAGACCTGGAACAATTCAACCTGGAGCAACCAGACCCAGAACATCCAGTCCTGGAGCAACCACTCCTGGAACACTCAGACCTGGTGCACCCAATCCTGGAACAATCAGGCCTGGAACAGTCCCTTCTATAACTGTGGAGAGGAATCTCTGCAGTCCTGCATGCAGTTCCAGCCAAATTCTCCTGCCAGTGACTTGGAGGCTGCCTTGGAAGCTGCTGGGGAAGGCCTTAATGTAATACAGCAGACCACTAGGTATTTTAGTACTCCACAAACCATGGATTTATTCCTAAACTACTCCATGAACATGCAACCTGAAGACGTGTGAggtacc

MUT-2 target sequence：

gctagcATGAGTGTGGATCCAGCTTGTCCCCAAAGCTTGCCTTGCTTTGAAGCATCCGACTGTAAAGAATCTTCACCTATGCCTGTGATTTGTGGGCCTGAAGAAAACTATCCATCCTTGCAAATGTCTTCTGCTGAGATGCCTCACACGGAGACTGTCTCTCCTCTTCCTTCCTCCATGGATCTGCTTATTCAGGACAGCCCTGATTCTTCCACCAGTCCCAAAGGCAAACAACCCACTTCTGCAGAGAAGAGTGTCGCAAAAAAGGAAGACAAGGTCCCGGTCAAGAAACAGAAGACCAGAACTGTGTTCTCTTCCACCCAGCTGTGTGTACTCAATGATAGATTTCAGAGACAGAAATACCTCAGCCTCCAGCAGATGCAAGAACTCTCCAACATCCTGAACCTCAGCTACAAACAGGTGAAGACCTGGTTCCAGAACCAGAGAATGAAATCTAAGAGGTGGCAGAAAAACAACTGGCCGAAGAATAGCAATGGTGTGACGCAGAAGGCCTCAGCACCTACCTACCCCAGCCTTTACTCTTCCTACCACCAGGGATGCCTGGTGAACCCGACTGGGAACCTTCCAATGTGGAGCgcaCAGACCTGGAACAATTCAACCTGGAGCAACCAGACCCAGAACATCCAGTCCTGGAGCAACCACTCCTGGAACACTCAGACCTGGTGCACCCAATCCTGGAACAATCAGGCCTGGAACAGTCCCTTCTATAACTGTGGAGAGGAATCTCTGCAGTCCTGCATGCAGTTCCAGCCAAATTCTCCTGCCAGTGACTTGGAGGCTGCCTTGGAAGCTGCTGGGGAAGGCCTTAATGTAATACAGCAGACCACTAGGTATTTTAGTACTCCACAAACCATGGATTTATTCCTAAACTACTCCATGAACATGCAACCTGAAGACGTGTGAggtacc

MUT-3 target sequence：

gctagcATGAGTGTGGATCCAGCTTGTCCCCAAAGCTTGCCTTGCTTTGAAGCATCCGACTGTAAAGAATCTTCACCTATGCCTGTGATTTGTGGGCCTGAAGAAAACTATCCATCCTTGCAAATGTCTTCTGCTGAGATGCCTCACACGGAGACTGTCTCTCCTCTTCCTTCCTCCATGGATCTGCTTATTCAGGACAGCCCTGATTCTTCCACCAGTCCCAAAGGCAAACAACCCACTTCTGCAGAGAAGAGTGTCGCAAAAAAGGAAGACAAGGTCCCGGTCAAGAAACAGAAGACCAGAACTGTGTTCTCTTCCACCCAGCTGTGTGTACTCAATGATAGATTTCAGAGACAGAAATACCTCAGCCTCCAGCAGATGCAAGAACTCTCCAACATCCTGAACCTCAGCTACAAACAGGTGAAGACCTGGTTCCAGAACCAGAGAATGAAATCTAAGAGGTGGCAGAAAAACAACTGGCCGAAGAATAGCAATGGTGTGACGCAGAAGGCCTCAGCACCTACCTACCCCAGCCTTTACTCTTCCTACCACCAGGGATGCCTGGTGAACCCGACTGGGAACCTTCCAATGTGGAGCAACCAGACCTGGgccAATTCAACCTGGAGCAACCAGACCCAGAACATCCAGTCCTGGAGCAACCACTCCTGGAACACTCAGACCTGGTGCACCCAATCCTGGAACAATCAGGCCTGGAACAGTCCCTTCTATAACTGTGGAGAGGAATCTCTGCAGTCCTGCATGCAGTTCCAGCCAAATTCTCCTGCCAGTGACTTGGAGGCTGCCTTGGAAGCTGCTGGGGAAGGCCTTAATGTAATACAGCAGACCACTAGGTATTTTAGTACTCCACAAACCATGGATTTATTCCTAAACTACTCCATGAACATGCAACCTGAAGACGTGTGAggtacc

MUT-4 target sequence：

gctagcATGAGTGTGGATCCAGCTTGTCCCCAAAGCTTGCCTTGCTTTGAAGCATCCGACTGTAAAGAATCTTCACCTATGCCTGTGATTTGTGGGCCTGAAGAAAACTATCCATCCTTGCAAATGTCTTCTGCTGAGATGCCTCACACGGAGACTGTCTCTCCTCTTCCTTCCTCCATGGATCTGCTTATTCAGGACAGCCCTGATTCTTCCACCAGTCCCAAAGGCAAACAACCCACTTCTGCAGAGAAGAGTGTCGCAAAAAAGGAAGACAAGGTCCCGGTCAAGAAACAGAAGACCAGAACTGTGTTCTCTTCCACCCAGCTGTGTGTACTCAATGATAGATTTCAGAGACAGAAATACCTCAGCCTCCAGCAGATGCAAGAACTCTCCAACATCCTGAACCTCAGCTACAAACAGGTGAAGACCTGGTTCCAGAACCAGAGAATGAAATCTAAGAGGTGGCAGAAAAACAACTGGCCGAAGAATAGCAATGGTGTGACGCAGAAGGCCTCAGCACCTACCTACCCCAGCCTTTACTCTTCCTACCACCAGGGATGCCTGGTGAACCCGACTGGGAACCTTCCAATGTGGAGCAACCAGACCTGGAACgctTCAACCTGGAGCAACCAGACCCAGAACATCCAGTCCTGGAGCAACCACTCCTGGAACACTCAGACCTGGTGCACCCAATCCTGGAACAATCAGGCCTGGAACAGTCCCTTCTATAACTGTGGAGAGGAATCTCTGCAGTCCTGCATGCAGTTCCAGCCAAATTCTCCTGCCAGTGACTTGGAGGCTGCCTTGGAAGCTGCTGGGGAAGGCCTTAATGTAATACAGCAGACCACTAGGTATTTTAGTACTCCACAAACCATGGATTTATTCCTAAACTACTCCATGAACATGCAACCTGAAGACGTGTGAggtacc

MUT-5 target sequence：

gctagcATGAGTGTGGATCCAGCTTGTCCCCAAAGCTTGCCTTGCTTTGAAGCATCCGACTGTAAAGAATCTTCACCTATGCCTGTGATTTGTGGGCCTGAAGAAAACTATCCATCCTTGCAAATGTCTTCTGCTGAGATGCCTCACACGGAGACTGTCTCTCCTCTTCCTTCCTCCATGGATCTGCTTATTCAGGACAGCCCTGATTCTTCCACCAGTCCCAAAGGCAAACAACCCACTTCTGCAGAGAAGAGTGTCGCAAAAAAGGAAGACAAGGTCCCGGTCAAGAAACAGAAGACCAGAACTGTGTTCTCTTCCACCCAGCTGTGTGTACTCAATGATAGATTTCAGAGACAGAAATACCTCAGCCTCCAGCAGATGCAAGAACTCTCCAACATCCTGAACCTCAGCTACAAACAGGTGAAGACCTGGTTCCAGAACCAGAGAATGAAATCTAAGAGGTGGCAGAAAAACAACTGGCCGAAGAATAGCAATGGTGTGACGCAGAAGGCCTCAGCACCTACCTACCCCAGCCTTTACTCTTCCTACCACCAGGGATGCCTGGTGAACCCGACTGGGAACCTTCCAATGTGGAGCAACCAGACCTGGAACAATTCAACCTGGAGCgccCAGACCCAGAACATCCAGTCCTGGAGCAACCACTCCTGGAACACTCAGACCTGGTGCACCCAATCCTGGAACAATCAGGCCTGGAACAGTCCCTTCTATAACTGTGGAGAGGAATCTCTGCAGTCCTGCATGCAGTTCCAGCCAAATTCTCCTGCCAGTGACTTGGAGGCTGCCTTGGAAGCTGCTGGGGAAGGCCTTAATGTAATACAGCAGACCACTAGGTATTTTAGTACTCCACAAACCATGGATTTATTCCTAAACTACTCCATGAACATGCAACCTGAAGACGTGTGAggtacc

MUT-6 target sequence：

gctagcATGAGTGTGGATCCAGCTTGTCCCCAAAGCTTGCCTTGCTTTGAAGCATCCGACTGTAAAGAATCTTCACCTATGCCTGTGATTTGTGGGCCTGAAGAAAACTATCCATCCTTGCAAATGTCTTCTGCTGAGATGCCTCACACGGAGACTGTCTCTCCTCTTCCTTCCTCCATGGATCTGCTTATTCAGGACAGCCCTGATTCTTCCACCAGTCCCAAAGGCAAACAACCCACTTCTGCAGAGAAGAGTGTCGCAAAAAAGGAAGACAAGGTCCCGGTCAAGAAACAGAAGACCAGAACTGTGTTCTCTTCCACCCAGCTGTGTGTACTCAATGATAGATTTCAGAGACAGAAATACCTCAGCCTCCAGCAGATGCAAGAACTCTCCAACATCCTGAACCTCAGCTACAAACAGGTGAAGACCTGGTTCCAGAACCAGAGAATGAAATCTAAGAGGTGGCAGAAAAACAACTGGCCGAAGAATAGCAATGGTGTGACGCAGAAGGCCTCAGCACCTACCTACCCCAGCCTTTACTCTTCCTACCACCAGGGATGCCTGGTGAACCCGACTGGGAACCTTCCAATGTGGAGCAACCAGACCTGGAACAATTCAACCTGGAGCAACCAGACCCAGAACATCCAGTCCTGGAGCgccCACTCCTGGAACACTCAGACCTGGTGCACCCAATCCTGGAACAATCAGGCCTGGAACAGTCCCTTCTATAACTGTGGAGAGGAATCTCTGCAGTCCTGCATGCAGTTCCAGCCAAATTCTCCTGCCAGTGACTTGGAGGCTGCCTTGGAAGCTGCTGGGGAAGGCCTTAATGTAATACAGCAGACCACTAGGTATTTTAGTACTCCACAAACCATGGATTTATTCCTAAACTACTCCATGAACATGCAACCTGAAGACGTGTGAggtacc

MUT-7 target sequence：

gctagcATGAGTGTGGATCCAGCTTGTCCCCAAAGCTTGCCTTGCTTTGAAGCATCCGACTGTAAAGAATCTTCACCTATGCCTGTGATTTGTGGGCCTGAAGAAAACTATCCATCCTTGCAAATGTCTTCTGCTGAGATGCCTCACACGGAGACTGTCTCTCCTCTTCCTTCCTCCATGGATCTGCTTATTCAGGACAGCCCTGATTCTTCCACCAGTCCCAAAGGCAAACAACCCACTTCTGCAGAGAAGAGTGTCGCAAAAAAGGAAGACAAGGTCCCGGTCAAGAAACAGAAGACCAGAACTGTGTTCTCTTCCACCCAGCTGTGTGTACTCAATGATAGATTTCAGAGACAGAAATACCTCAGCCTCCAGCAGATGCAAGAACTCTCCAACATCCTGAACCTCAGCTACAAACAGGTGAAGACCTGGTTCCAGAACCAGAGAATGAAATCTAAGAGGTGGCAGAAAAACAACTGGCCGAAGAATAGCAATGGTGTGACGCAGAAGGCCTCAGCACCTACCTACCCCAGCCTTTACTCTTCCTACCACCAGGGATGCCTGGTGAACCCGACTGGGAACCTTCCAATGTGGAGCAACCAGACCTGGAACAATTCAACCTGGAGCAACCAGACCCAGAACATCCAGTCCTGGAGCAACCACTCCTGGAACACTCAGACCTGGTGCACCCAATCCTGGAACAATCAGGCCTGGAACAGTCCCTTCTATAACTGTGGAGAGGAATCTCTGCAGTCCTGCATGCAGTTCCAGCCAAATTCTCCTGCCAGTGACTTGGAGGCTGCCTTGGAAGCTGCTGGGGAAGGCCTTAATGTAATACAGCAGACCACTAGGTATTTTAGTACTCCACAAACCATGGATTTATTCCTAgccTACTCCATGAACATGCAACCTGAAGACGTGTGAggtacc
