# Supplementary material for: N-glycosylation of NANOG regulates stemness and apoptosis in colon cancer cells
Source: PLoS One. 2025 Nov 20;20(11):e0336779. doi: 10.1371/journal.pone.0336779 (PMC12633897; doi:10.1371/journal.pone.0336779)
Supplement: S2 File — (DOCX) [file pone.0336779.s002.docx]

MTT experiment

HCT116 stem cells


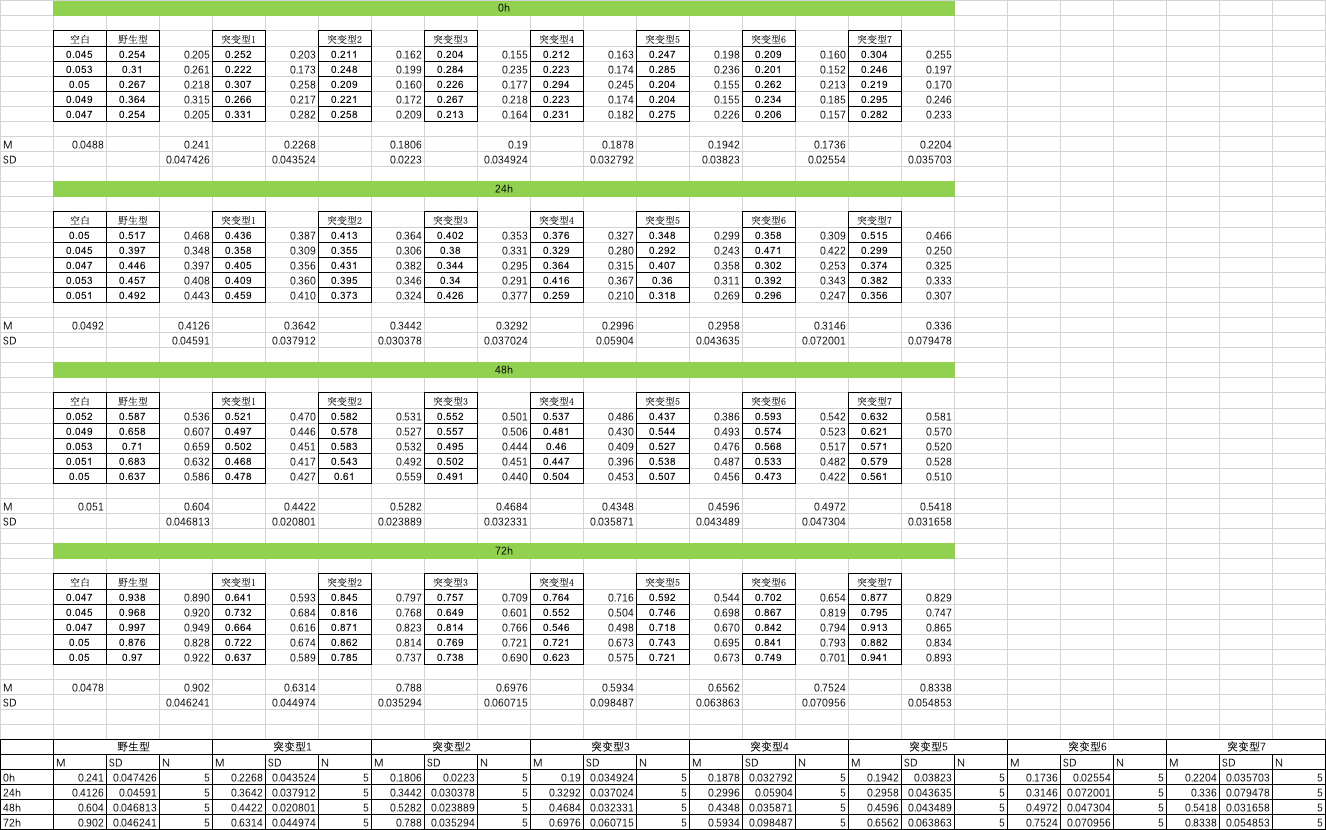


LoVo stem cells


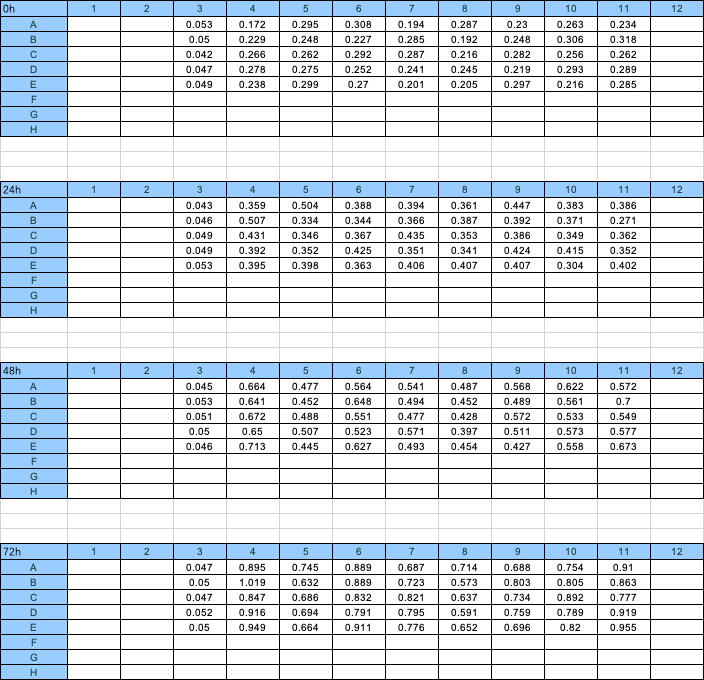


EdU flow cytometry

HCT116 stem cells

| WT | MUT-1 | MUT-2 | MUT-3 | MUT-4 | MUT-5 | MUT-6 | MUT-7 |
| --- | --- | --- | --- | --- | --- | --- | --- |
| 47.5 | 5.77 | 21.6 | 12.2 | 8.16 | 5.42 | 10.4 | 34.4 |
| 49.2 | 5.57 | 14.3 | 5.69 | 4.97 | 8.17 | 10.1 | 32 |
| 50.2 | 10 | 15.7 | 9.48 | 5.75 | 6.59 | 8.84 | 33.4 |

LoVo stem cells

| WT | MUT-1 | MUT-2 | MUT-3 | MUT-4 | MUT-5 | MUT-6 | MUT-7 |
| --- | --- | --- | --- | --- | --- | --- | --- |
| 84.9 | 35.7 | 34.1 | 31.8 | 29.3 | 7.33 | 25.9 | 53.8 |
| 83 | 38.6 | 36.6 | 30.2 | 24.6 | 7.04 | 32.3 | 54.9 |
| 86.3 | 38.4 | 41.3 | 35.4 | 17.4 | 10.4 | 32.4 | 45.7 |

Stem cell sphere formation test

HCT116 stem cells

LoVo stem cells

Cell migration assay

| WT | MUT-1 | MUT-2 | MUT-3 | MUT-4 | MUT-5 | MUT-6 | MUT-7 |
| --- | --- | --- | --- | --- | --- | --- | --- |
| 632.21 | 183.69 | 369.6 | 261.69 | 153.05 | 232.19 | 367.76 | 512.37 |
| 649.04 | 185.48 | 405.85 | 281.68 | 151.4 | 253.02 | 322.12 | 590.83 |
| 627.59 | 167.75 | 428.93 | 289.19 | 91.08 | 220.45 | 356.1 | 556.25 |
