# Supplementary material for: N-glycosylation of NANOG regulates stemness and apoptosis in colon cancer cells
Source: PLoS One. 2025 Nov 20;20(11):e0336779. doi: 10.1371/journal.pone.0336779 (PMC12633897; doi:10.1371/journal.pone.0336779)

Figure2

NANOG in HCT166 cells

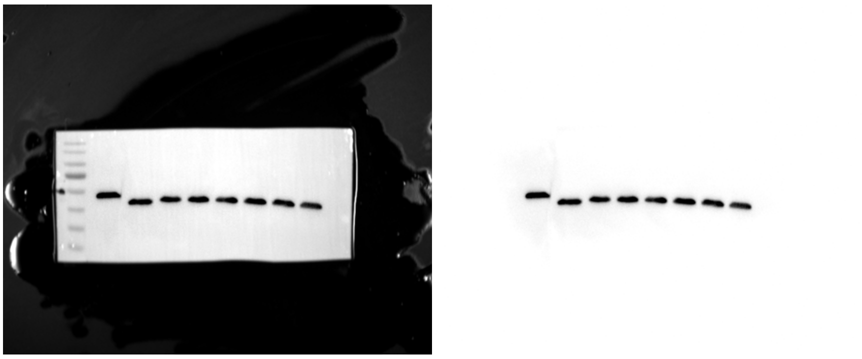

GAPDH in HCT166 cells

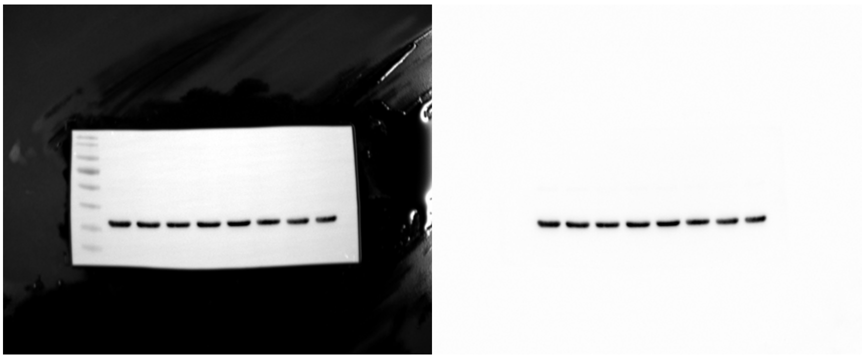

NANOG in LoVo cells

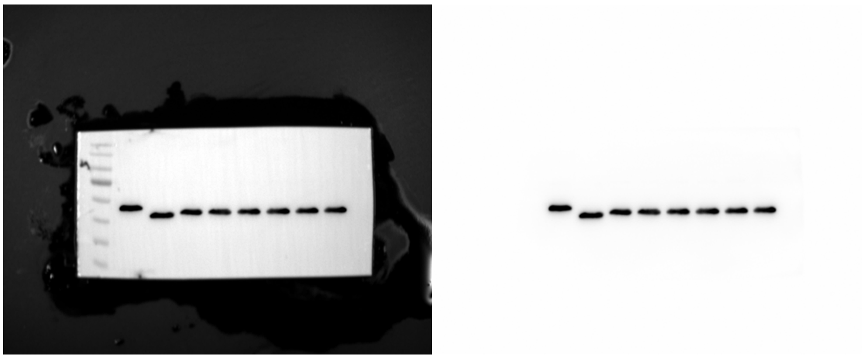

GAPDH in LoVo cells

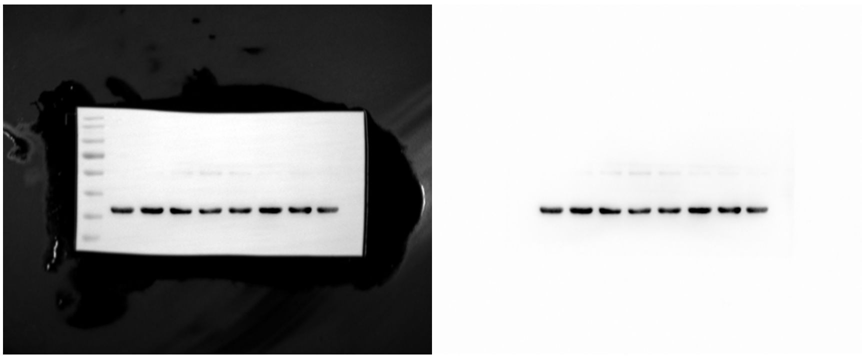

Figure8

BAX in HCT166 cells

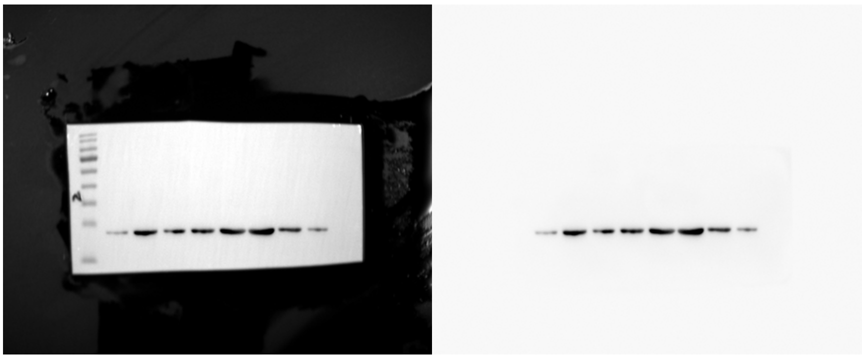

Bcl2 in HCT166 cells

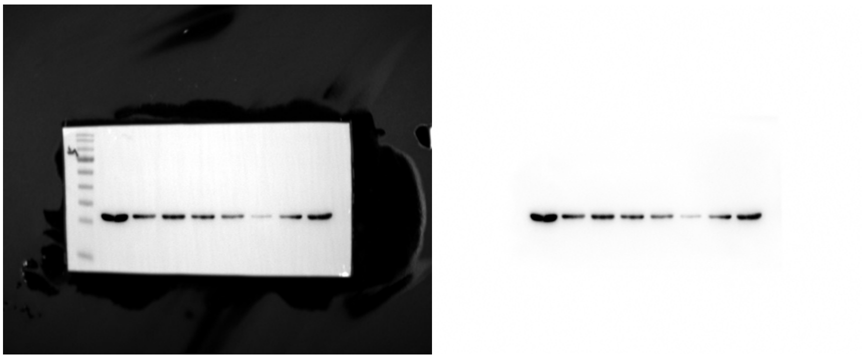

GAPDH in HCT166 cells

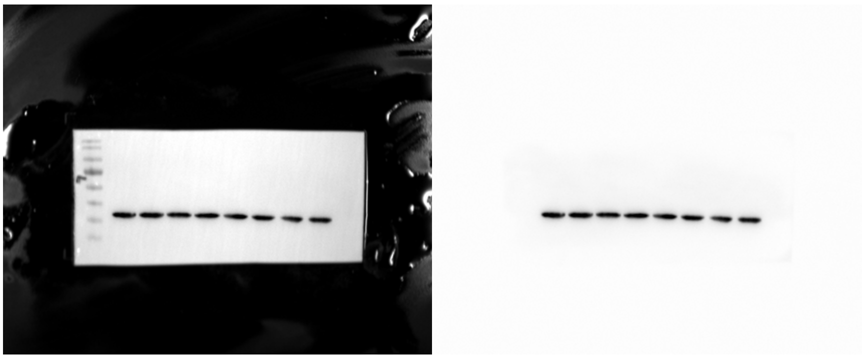

BAX in LoVo cells

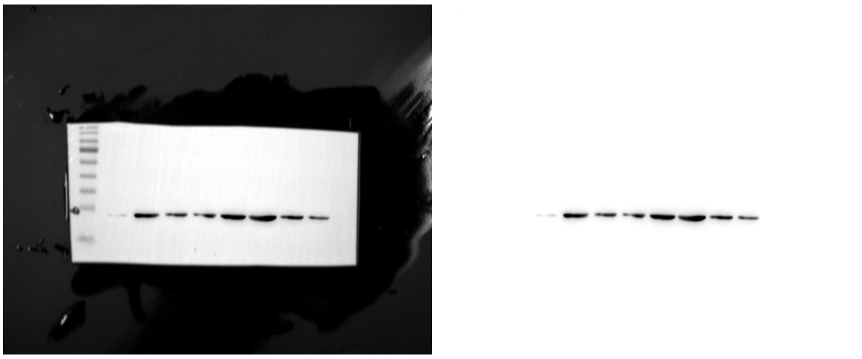

Bcl2 in LoVo cells

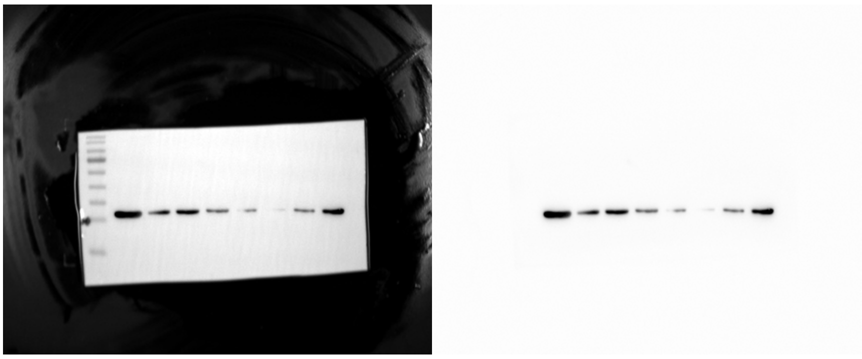

GAPDH in LoVo cells

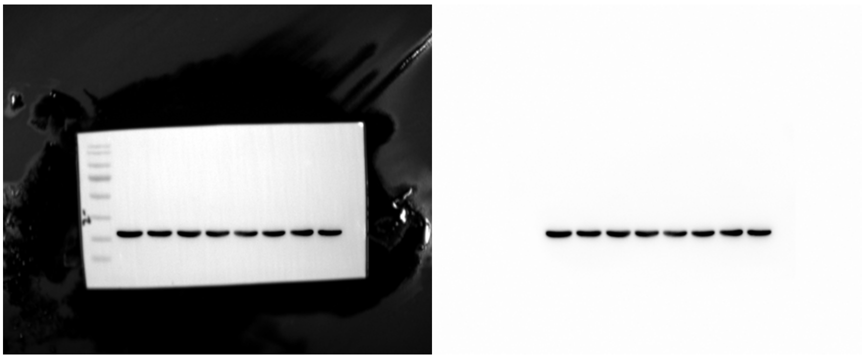

Supplement: S3 File — (PDF) [file pone.0336779.s003.pdf]
