# Supplementary material for: N-glycosylation of NANOG regulates stemness and apoptosis in colon cancer cells
Source: PLoS One. 2025 Nov 20;20(11):e0336779. doi: 10.1371/journal.pone.0336779 (PMC12633897; doi:10.1371/journal.pone.0336779)
Supplement: Attachment — Submitted filename: renamed_a7b53.docx [file pone.0336779.s006.docx]

1. Please ensure that your manuscript meets PLOS ONE's style requirements, including those for file naming.

Response: Thanks for the suggestions. we misunderstood the format requirements and we’ve revised the manuscript format to meet PLOS ONE’s style requirements as possible.

2. We noticed you have some minor occurrence of overlapping text with the following previous publication(s).

**Response:** Thanks for the suggestions. During the writing of the manuscript, some content cited from references was missing proper citations. In the revised version, we have rephrased some of the text and supplemented the citations of the references.

3. Please state what role the funders took in the study.

**Response:** Thanks for the suggestions. The role of the funder is to design the study and make the decision to publish. We may omitted this information in the online submission system.

4. Please remove any funding-related text from the manuscript and let us know how you would like to update your Funding Statement.

**Response:** Thanks for the suggestions. We removed the funding-related text from the manuscript, and it need no more update.

5. Please ensure that you have an ORCID iD and that it is validated in Editorial Manager.

**Response:** Thanks for the suggestions. We validated the ORCID in Editorial Manager.

6. In your cover letter, please note whether your blot/gel image data are in Supporting Information or posted at a public data repository.

**Response:** Thanks for the suggestions. We attached the original blot/gel image data in the Supporting Information file, which named WB original pictures, and uploaded with other files in the online submission system.

7. Please include a separate caption for each figure in your manuscript.

**Response:** Thanks for the suggestions. During the submission process, we may have misunderstood the submission requirements and uploaded the figures along with their corresponding textual descriptions as a separate file (named "Figures") to the online submission system. Another editor also pointed out the issue of missing figures and tables. In this major revision, we will incorporate the key figures/tables along with their corresponding descriptions into the “revised manuscript with track changes”. If this still does not meet the submission format requirements, please inform us promptly so that we can make further corrections.

Major Comments:

1. The current title, "N-glycosylation regulates NANOG affects stem cell characteristics and apoptosis of colon cancer cells," is grammatically awkward, lengthy, and unclear. It should be revised for precision and conciseness.

**Response:** Thanks for the suggestions. We’ve changed the title as “N-glycosylation of NANOG regulates stemness and apoptosis in colon cancer cells”due to the comments.

2. Missing Figures (Very Critical) problem.

**Response:** Thanks for the suggestions. During the submission process, we may have misunderstood the submission requirements and uploaded the figures along with their corresponding textual descriptions as a separate file (named "Figures") to the online submission system. In this major revision, we will incorporate the key figures/tables along with their corresponding descriptions into the “revised manuscript with track changes”. If this still does not meet the submission format requirements, please inform us promptly so that we can make further corrections.

3. There is an inconsistency in the manuscript regarding the HCT166/HCT116 cell line.

**Response:** Thanks for the suggestions. In this study, In this study, two colon cancer cell lines, HCT116 and LoVo colon cancer cells, were utilized for the relevant experiments. We sincerely apologize for mistakenly writing HCT116 as HCT166 in some passages. We have corrected this error at the corresponding positions.

4. There is a lack of detailed Western Blot Protocol.

**Response:** Thanks for the suggestions. We sincerely apologize that during the manuscript writing process, we omitted the operational procedures for the Western blot (WB) experiments, which have now been supplemented in the revised manuscript.

5. Apoptosis Results Description is ambiguous: In Section 3.6, the statement "the apoptosis levels of HCT116 colon cancer stem cells and LoVo colon cancer stem cells after transfection of 7 MUT plasmids of NANOG protein decreased (Figure 8)" needs careful rephrasing for clarity. If N-glycosylation is stated to "promote apoptosis" (as in the abstract and section title), then the mutation/removal of glycosylation sites (via MUT plasmids) should lead to decreased apoptosis, which aligns with the finding. However, the phrasing "N-glycosylation participates in regulating NANOG protein to promote apoptosis" and then showing decreased apoptosis in mutants can be confusing. Consider phrasing like "N-glycosylation is required for NANOG protein to promote apoptosis" or "N-glycosylation enhances NANOG-mediated apoptosis."

**Response:** Thanks for the suggestions. The mutation/removal of glycosylation sites was achieved via transfection of mutant plasmids. Our findings revealed that the elimination of NANOG glycosylation led to a reduction in the apoptosis level of cancer stem cells. We appreciate the editor's comments and have revised the subheading to convey the information more accurately.

Minor points:

1. The introduction mentions screening for "N-glycosylase" expression but could benefit from explicitly naming OST-A and OST-B earlier, as these are the specific enzymes identified and discussed in the results.

**Response:** Thanks for the suggestions. In the Introduction section, we first introduce the NANOG protein, followed by a discussion on the impact of aberrant N-glycosylation on NANOG-mediated regulation of cancer stem cells. Subsequently, we cite literature reports indicating that human OST complexes, OST-A (containing STT3A) and OST-B (containing STT3B), are involved in N-linked glycosylation of proteins in the endoplasmic reticulum. We believe that explicitly mentioning OST-A and OST-B at an earlier stage may slightly compromise the current logical flow and coherence of the manuscript. We welcome any further detailed suggestions from the editor and are happy to make revisions accordingly.

2. While the general mutation strategy is stated (N-X-T(S) to A-X-T(S)), more specific information about the exact mutation sites for each of the seven mutants (MUT-1 to MUT-7) would significantly enhance the reproducibility of the study.

**Response:** Thanks for the suggestions. We will include additional information on the 7 mutants in the supplementary files, including the exacttarget sequence of each mutant.

3. Lack of Rationale for CD133+ Cell Selection: why CD133+ cells were selected as the primary stem cell population for colon cancer or provide a relevant citation to support this choice. **Response:** Thanks for your question. CD133 (prominin-1) was selected as a marker for isolating colon cancer stem cells (CSCs) based on extensive prior evidence demonstrating its association with stemness properties in colorectal cancer. In this article(doi:10.1517/14728222.2012.667404), the researcher demonstrated that CD133⁺ cells isolated from colon cancer specimens (accounting for 2.5% of total cells) consistently formed xenograft tumors in immunodeficient mice. Moreover, with serial in vivo passages, CD133⁺ cells exhibited progressively enhanced aggressiveness (manifested as accelerated tumor growth and an increased proportion of CD133⁺ cells in subsequent generations of tumors), whereas CD133⁻ cells lacked this capability. The core viewpoint of another literature(doi:10.4252/wjsc.v11.i11.920) indicates that stemness in colon cancer is "a reversible epigenetic state" rather than a fixed genetic identity, and the expression of CD133 is precisely regulated by canonical epigenetic mechanisms, which aligns with the "plasticity" characteristic of stem cells. Besides, it also pointed out that the standalone use of CD133 for identifying colorectal cancer stem cells (CR-CSCs) has limitations, but its combination with other markers (CD44, CD166) significantly improves specificity, with CD133 serving as the core "anchor marker" in such combinatorial strategies. Multiple studies have shown that CD133+ subpopulations in colon cancer exhibit enhanced self-renewal capacity, tumorigenicity in xenograft models, and resistance to chemotherapy—hallmarks of CSCs.
4. Provide additional details or a reference for the "WZ_Camera" software used for measurements, if it is not a widely recognized commercial software.

**Response:** Thanks for the suggestions. WZ_Camera is a domestically developed image measurement software with a more concise and user-friendly interface compared to widely recognized commercial software. The images in the manuscript were also analyzed using Image J, and the resulting data were consistent.
